# Nuremberg Letterbooks: A Multi-Transcriptional Dataset of Early 15th Century Manuscripts for Document Analysis

**DOI:** 10.1038/s41597-025-05144-z

**Published:** 2025-05-17

**Authors:** Martin Mayr, Julian Krenz, Katharina Neumeier, Anna Bub, Simon Bürcky, Nina Brolich, Klaus Herbers, Mechthild Habermann, Peter Fleischmann, Andreas Maier, Vincent Christlein

**Affiliations:** 1 Pattern Recognition Lab, Friedrich-Alexander-Universität Erlangen-Nürnberg, Erlangen, 91058 Germany; 2 Senior Fellow of Medieval History, Friedrich-Alexander-Universität Erlangen-Nürnberg, Erlangen, 91054 Germany; 3 Department of German Linguistics, Friedrich-Alexander-Universität Erlangen-Nürnberg, Erlangen, 91054 Germany; 4 Chair of Regional History of Bavaria and Franconia, Friedrich-Alexander-Universität Erlangen-Nürnberg, Erlangen, 91054 Germany

**Keywords:** History, Scientific data

## Abstract

Most datasets in the field of document analysis utilize highly standardized labels, which, while simplifying specific tasks, often produce outputs that are not directly applicable to humanities research. In contrast, the Nuremberg Letterbooks dataset, which comprises historical documents from the early 15th century, addresses this gap by providing multiple types of transcriptions and accompanying metadata. This approach allows for developing methods that are more closely aligned with the needs of the humanities. The dataset includes 4 books containing 1711 labeled pages written by 10 scribes. Three types of transcriptions are provided for handwritten text recognition: Basic, diplomatic, and regularized. For the latter two, versions with and without expanded abbreviations are also available. A combination of letter ID and writer ID supports writer identification due to changing writers within pages. Additionally, we provide metadata, including line bounding boxes and text regions. In the technical validation, we established baselines for various tasks, demonstrating data consistency and providing benchmarks for future research to build upon.

## Background & Summary

In historical document digitization and handwritten text recognition, a significant challenge lies in bridging the gap between merely scanning ancient manuscripts and truly accessing and understanding the corpus they present. While digitization has made these texts more available, it does not inherently make them comprehensible or usable for varied research purposes. The process of transitioning from a physical document to a digital corpus encompasses numerous complexities, especially in transcription and interpretation, which vary widely based on the research field and study objectives.

- Computer scientists, for instance, often work with basic transcriptions of documents. These transcriptions are simplified versions tailored for training text recognition models.
- In contrast, German studies require transcriptions that are as close as possible to the original text, capturing its visual features and nuances. This level of detail is crucial for studies focused on linguistic and cultural contexts.
- Historians, on the other hand, lean towards regularized versions of texts where abbreviations are resolved and special characters are normalized to suit the reading habits of the contemporary audience. This approach facilitates content analysis and interpretation, making historical texts more accessible and understandable. These regularized versions often culminate in the creation of scholarly editions.

The Nuremberg Letterbooks illustrate the critical need for varied transcription methods in document analysis. Historically, they were used to record the outgoing letters (correspondences) of Nuremberg’s small council to other cities and individuals. The topics of the correspondence range from everyday economic or legal matters of individual citizens to discussions of imperial politics with the kings or other major cities. These books span multiple centuries, from 1404 to 1738, and are contained in 358 volumes (https://www.gda.bayern.de/service/findmitteldatenbank/Kapitel/1a7dcb6f-0973-4a4b-bbbb-4286460e45d4). However, some books are missing, as indicated by the earliest recovered book (Staatsarchiv Nürnberg, Reichsstadt Nürnberg, Ratskanzlei, Briefbücher des Inneren Rates 1), which is labeled with “VII” on its front page. The first recovered volume covers the period from March 21, 1404, to February 8, 1408. Research on this book led to a historical edition^1^, which is not included in this dataset due to differences in data creation methods and a primary focus on historians. Building on this initial digitization effort, an interdisciplinary DFG-funded project worked on accessing and analyzing the next four books (Staatsarchiv Nürnberg, Reichsstadt Nürnberg, Ratskanzlei, Briefbücher des Inneren Rates 2-5, covering the period from February 7, 1408, to March 31, 1423). The project’s goal was to make the data usable for researchers across multiple disciplines (History, German Studies, and Computer Science) and to develop algorithms for faster transcription and metadata extraction from historical documents of this type. As part of this project, three types of annotations and multiple meta data were created for the four successive books:

1. Basic transcriptions primarily intended for automatic text recognition, often serving as the default^2–5^.
2. Diplomatic transcriptions tailored for German studies, available with or without resolved abbreviations, depending on the use case.
3. Regularized transcriptions designed for historians.
4. Text regions for layout analysis of letters (records of correspondence).
5. Line bounding boxes for line segmentation.
6. Main writer label per letter for writer retrieval tasks.

The primary audience for this paper is researchers in the field of document analysis. Given the diverse types of annotations available, new approaches can be developed to bridge the gap between the outputs of current document analysis systems and the actual needs of humanities scholars. The goal is to minimize the manual effort required for historians and linguists to effectively work with automatically generated data.

The remainder of this paper is structured into three main sections. The Methods section details the data acquisition and processing pipeline, including manual labeling of transcriptions and metadata as well as repeated semi-automated error detection and correction. The Data Records section explains where the data can be downloaded and provides further information about the dataset. Finally, the Technical Validation section demonstrates the dataset’s high consistency and offers a baseline for future research approaches.

## Methods

**Fig. 1:**
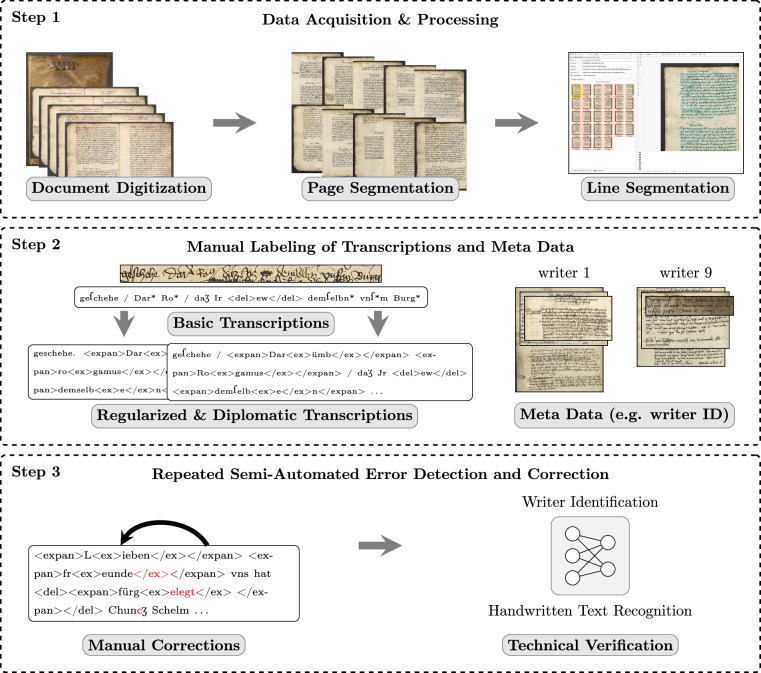
Step 1 describes data acquisition and preprocessing. The pages of the scanned documents are separated, with a subsequent line segmentation. In step 2, the transcriptions and meta data are manually labeled. The created basic transcriptions are used as a foundation for the regularized and diplomatic text versions. Simultaneously, the meta data, like writer IDs, are marked. In step 3, manual corrections are made, and the produced data is analyzed for technical validation.

Figure 1 outlines our study’s methodology, broken down into (1) data acquisition and processing, (2) manual labeling of transcriptions and meta data, and (3) repeated semi-automated error detection and correction.

### Data Acquistion and Processing

The goal of this stage is to extract the segmented lines from historical documents. To achieve this, the process followed three main steps: (1) scanning the documents, (2) splitting the pages, and (3) segmenting the lines. The documents were first scanned in color at 300 DPI as double-page spreads. Then, each double-page scan was semi-automatically segmented to isolate individual pages. To achieve accurate segmentation, we employed a Sobel kernel^6^ in the vertical direction to compute gradients, highlighting the edges within the image. These gradients were then summed along the vertical direction and multiplied by a Gaussian curve across the width. The focus on the center regions of the double scan is necessary due to false classifications of the borders of the text blocks as page breaks and the prior knowledge that each page’s boundary is generally located near the center of the scan. This initial segmentation process was further refined by applying small rotations to the image, ultimately selecting the highest gradient activation orientation. Each page was manually checked after the automated segmentation process because an error in this early stage would have propagated throughout all successive stages. After obtaining single pages, we utilized the line segmentation feature CITLab advanced^7^ of Transkribus (https://www.transkribus.org/), a specialist text recognition and transcription tool. It provided a solid foundation for the baselines and polygons of the text lines. However, strike-throughs and other uncommon text formatting often led to misclassifications, which were adjusted in the next step.

### Manual Labeling of Transcriptions and Meta Data

Once the lines were segmented, experts in German studies and historians, who specialized in documents from this era, created the basic transcription. Simultaneously to the transcription process, manual adjustments to the bounding boxes were made if necessary. Subsequently, we extended the basic transcriptions to create a diplomatic version, commonly used in German Studies, through a semi-automatic process. In the basic transcription, abbreviations were indicated with an asterisk (*) symbol, which was converted into <expan> tags surrounding the abbreviated word, while <ex></ex> was placed at the position of the asterisk. Additionally, we automatically integrated known elements, such as specific symbols, into the transcription. Experts in German Studies then refined the diplomatic transcription by adding details that closely reflect the original text while preserving its unique features and stylistic elements. As part of this process, they manually resolved abbreviations – sometimes with the assistance of historian experts – to ensure accurate interpretation. These expansions were then added to the <ex> tags.

Building upon the diplomatic version, we created a regularized version of the text through a semi-automatic process, utilizing both the basic and diplomatic transcriptions as a foundation. This process included modifications such as converting special symbols into contemporary characters, for example, replacing the long “s” with the modern “s” to enhance readability. Furthermore, elements typically excluded from regularized transcriptions, such as deletions, were automatically removed. The text was also refined by integrating marginal notes and interlinear additions, when possible, while eliminating irrelevant style information from within the tags. Finally, experts not only reviewed and corrected all modifications but also transformed the text to improve readability for a contemporary audience

During this process, additional information was captured, such as the manually adjusted line coordinates. The generated PAGE XML^8^ files also include the text regions for each letter (record of correspondence), determined based on the line coordinates and the expertise of the scholars who identified where each correspondence begins and ends. Note that these letters can vary in length, ranging from just a few lines on a page alongside other letters, to spanning multiple pages. Furthermore, our humanities experts analyzed the writing style to assign writer IDs to each letter (record of correspondence) in the documents. They focused specifically on the main writer, as in some letters, other historical writers had added the original dating retrospectively.

### Repeated Semi-Automated Error Detection and Correction

Creating such a large dataset with various nuanced types of information inevitably introduces the potential for human errors. To mitigate this, we implemented multiple rounds of corrections through technical verification. This process was applied to the fine-grained transcription types (basic and diplomatic, with expanded abbreviations) and to the writer IDs.

**Fig. 2:**
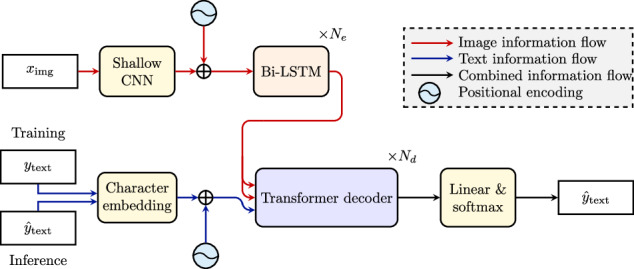
Overview of the Handwritten Text Recognition model. Red arrows show the image information flow, blue arrows show the text information flow, and black arrows show the combined information flow. The architecture is a combination of a shallow CNN and a transformer.

To identify transcription errors, experts compared the initial transcriptions with those generated automatically, assigning the correct labels. The automatic transcriptions were produced by training the same Handwritten Text Recognition (HTR) model multiple times using a leave-one-book-out strategy, where each book served once as the test set. To avoid overfitting to incorrect labels, only the test set outputs were used for the comparison. This process was applied to both the basic transcriptions and the diplomatic transcriptions with expanded abbreviations. For the train-validation split, we followed a set rule: if book 4 is not the test set, it is always used for validation. Otherwise, book 2 serves as the validation set, while the remaining two books are used for training. This ensures consistent evaluation and preserves training data by selecting the smallest books for validation. To support generalization, each book was assigned to only one split. Book 4 was preferred for validation due to its clean transcriptions after the initial manual pass. We kept the splits unchanged to maintain consistency throughout the semi-automatic error detection and correction. The same splits were also used for the final technical validation (see Table 3). As HTR architecture we employed a simplified sequence-to-sequence model, based on established approaches^9,10^. The model architecture, depicted in Fig. 2, consists of a shallow CNN^9,10^ followed by a bidirectional LSTM^11^ encoder and a transformer decoder^12^. The CNN extracts visual features from the image and feeds them as an input sequence to the LSTM encoder. To ensure compatibility with the encoder, the 2D feature map produced by the CNN is concatenated along the height dimension, converting it into the appropriate sequential input format. Based on the previous tokens and the encoded image features, the decoder predicts the output sequence auto-regressively. For fast training, teacher forcing with a noise probability of 0.1 was applied. Regarding the model parameters, we used three bidrectional LSTM layers, each with a hidden size of 256 and a dropout rate of 0.5 for regularization. The transformer decoder has a model dimension of 256, feed-forward dimension of 512, two decoder layers, and a dropout rate of 0.1. We applied a learning rate of 2 ⋅ 10^−4^, 4096 warm-up steps, and a reduce-on-plateau learning rate scheduler with a patience of 70. Label smoothing of 0.4 was used to improve generalization. For more details, we refer to the publicly available implementation: https://github.com/M4rt1nM4yr/letterbooks_text_verification.

Additionally, to identify wrongly labeled writer IDs, we utilized a widely recognized writer retrieval pipeline, outlined by Christlein *et al*.^13–15^, to find mismatches between labels and the clustered writers. Similar to the correction process of the transcriptions, for every correction iteration, we conducted multiple training runs based on the four books used for testing. Despite the approach being unsupervised, we exclude the test book from the training process because the clustering is based on features that could potentially overfit to specific input patterns. The approach consists of several steps. First, the input images are binarized using Otsu thresholding^16^. For retrieving local feature vectors, RootSIFT descriptors^17^ were computed from SIFT keypoints^18^, which were then PCA-whitened and dimensionality-reduced^14^ to 64 dimensions. The global feature vector is the result of a multi-VLAD approach^19^, where multiple VLAD encodings (with a multitude of 5) are concatenated, and the number of clusters for each VLAD encoding is set to 100. For better generalization, the global feature representation is again jointly whitened and dimensionality-reduced to 512 dimensions using PCA^13^. For more details, we made the implementation available on GitHub: https://github.com/M4rt1nM4yr/letterbooks_writer_verification.

## Data Records

The Nuremberg Letterbooks dataset is publicly available and hosted on the CERN-supported open repository Zenodo^20^. Table 1 provides an overview of the different books. Basic transcriptions are annotated using the PAGE XML format, with each page in the dataset corresponding to a PAGE XML file that includes information such as line polygons (which define the text regions), writer IDs (which identify the main writers of the letter), and basic transcription. Additional PAGE XML files provide both diplomatic and regularized transcriptions. The diplomatic version is linked to line polygons and writer IDs, while the regularized version is not linked to individual lines but instead presents normalized text at the letter level. Overall, the dataset consists of 50 980 annotated lines with basic transcriptions and 48 322 lines with diplomatic transcriptions. The basic transcriptions comprise 493 422 words with 32 707 word classes in total, showing variations in spelling. Additionally, the main writer of each letter was identified across all four books. In total, these books include 10 different writers. An overview of the writer distribution based on the number of correspondences is provided in Table 2. To facilitate access to the data, we have made our data loading and models available in separate GitHub repositories.

**Table 1:** Overview of the dataset, presenting the number of pages and lines for four historical books in both basic and diplomatic transcriptions. For the basic transcription, the table also includes the number of words and unique word classes. The total row provides a summary across all books.

	# of pages	# of lines	# of words	# of word classes	# of lines
book 2	256	8426	73468	9071	7997
book 3	548	16183	160297	15088	15471
book 4	290	8439	84255	8761	8134
book 5	617	17932	175402	15448	16720
total	1711	50980	493422	32707	48322

**Table 2:** The table shows the number of letters (correspondences) attributed to each writer in the respective books.

	Writers										
	W1	W2	W6	W7	W8	W9	W10	W11	W12	W13	total
book 2	247	46	52	125	1	—	—	—	—	—	471
book 3	348	75	76	494	74	—	—	—	—	—	1067
book 4	138	8	—	—	36	375	—	—	—	1	558
book 5	302	2	—	—	—	764	13	6	4	—	1091
total	1035	131	128	619	111	1139	13	6	4	1	3187

## Technical Validation

The technical validation serves two purposes: demonstrating the high data quality and establishing a baseline for other approaches working with this dataset. In the following, we present the results of our models performing line-based text recognition on both the basic and diplomatic transcriptions, including an ablation study on how well the HTR system resolves abbreviations. Additionally, we establish a baseline for writer retrieval across the different books.

### Basic and Diplomatic Transcriptions

For the final validation, we retrained the HTR model, as described in section “Repeated Semi-Automated Error Detection and Correction”, on basic transcriptions, diplomatic transcriptions without abbreviations, and diplomatic transcriptions with abbreviations. The same model parameters and hyperparameters were applied for training.

As evaluation metrics, we used the commonly applied Character Error Rate (CER) and Word Error Rate (WER) to measure the performance of HTR tasks^21,22^. It is important to note that words are separated solely by the *space* symbol; punctuation marks are included in the metric and may be present within words. Additionally, we assessed the accuracy of the automatically expanded abbreviations by introducing Abbreviation Error Rate (AER), which is defined as:

1 $$\,{\rm{AER}}=\frac{{\rm{\#\; of\; false\; abbreviations}}}{{\rm{\#\; of\; all\; abbreviations}}}\ .$$

We chose to measure if the complete expanded abbreviation is correct, including the position of the surrounding tags. Note that every part of the expanded abbreviation must be correctly predicted for a correct recognition.

Due to varying writer distributions, we adjusted the training, evaluation, and test splits, rerunning the HTR for each configuration. We assessed the HTR output against three versions of transcription: the basic transcription (Table 3), the diplomatic transcription without expanded abbreviations (Table 4), and the diplomatic transcription with expanded abbreviations (Table 5). For basic transcription, using book 4 for testing resulted in the lowest CER and WER. Book 2 and book 3, as test sets, performed similarly. However, book 5 had the highest CER and WER with 7.06% and 24.09%, respectively. Based on the poorer validation results, we hypothesize that the diverse distribution of writers in book 5 is crucial for achieving very good recognition results in the other books.

**Table 3:** HTR results on basic transcriptions. Results are given in percent [%].

Train sets	Validation set	Test set	CER_val_	WER_val_	CER_test_	WER_test_
book 3, book 5	book 4	book 2	2.64	9.80	3.49	11.65
book 2, book 5	book 4	book 3	2.98	10.91	3.71	12.25
book 3, book 5	book 2	book 4	3.49	11.64	2.88	10.61
book 2, book 3	book 4	book 5	5.88	20.73	7.06	24.09

**Table 4:** HTR results on diplomatic transcriptions. Results are given in percent [%].

Train sets	Validation set	Test set	CER_val_	WER_val_	CER_test_	WER_test_
book 3, book 5	book 4	book 2	2.27	8.49	4.14	14.74
book 2, book 5	book 4	book 3	2.47	9.13	3.94	13.47
book 3, book 5	book 2	book 4	4.06	14.42	2.41	9.01
book 2, book 3	book 4	book 5	6.61	23.56	7.11	25.40

**Table 5:** HTR results on diplomatic transcriptions with expanded abbreviations. Results are given in percent [%].

Train sets	Validation set	Test set	CER_val_	WER_val_	AER_val_	CER_test_	WER_test_	AER_test_
book 3, book 5	book 4	book 2	2.65	9.46	5.87	4.75	15.39	11.29
book 2, book 5	book 4	book 3	2.83	10.17	6.68	4.56	14.17	10.95
book 3, book 5	book 2	book 4	4.86	15.38	11.22	2.82	9.94	6.40
book 3, book 2	book 4	book 5	7.72	25.64	17.84	8.64	28.10	20.44

A similar trend can be seen in Table 4, which shows the results of the HTR model on the diplomatic transcriptions. As expected, due to the more complex data, on average the CER and WER are slightly increased compared to the basic transcriptions. Again, book 4 was the easiest to predict, and book 5 was the hardest. The CER and WER computed from the results of book 4 were even lower than those of the basic results. Table 5 depicts the recognition results with extended abbreviations. CER and WER dropped slightly because of longer output sequences, but they were still in a similar error range. Interestingly, the completion of the abbreviations worked very well. We hypothesize that the transformer decoder’s implicit language model receives enough samples from the training data to replace the abbreviation symbol with the appropriate text automatically.

### Writer Information

For the final validation of the writer information, we utilize the same approach as described in section “Repeated Semi-Automated Error Detection and Correction”.

To compute Mean Average Percision (mAP) and top-1, we used a leave-one-sample-out cross-validation, where each sample is picked as a query, and the remaining samples are ranked according to their similarity to the query. The mAP is computed from the ranks. We also report the top-1 accuracy. We give the results for each book. Despite the approach’s unsupervised nature, we split the test data from the train data.

Table 6 gives the results for a book-wise 4-fold-cross validation. The top-1 scores were almost perfect. However, when using book 3 for testing, mAP dropped below 90 %. The best mAP was achieved for book 5 with 96.4 % Also, for using all books as a basis for applying the unsupervised training method and also for testing, the results were still very good despite the more extensive test set with a top-1 score and mAP of 99.3 % and 85.7 %, respectively.

**Table 6:** Writer identification results. Results are given in percent [%].

Train sets	Test set	Top—1	mAP
book 3, book 4, book 5	book 2	99.6	92.8
book 2, book 4, book 5	book 3	99.3	88.3
book 2, book 3, book 5	book 4	99.5	93.8
book 2, book 3, book 4	book 5	99.6	96.4
All books	All books	99.3	85.7

**Fig. 3:**
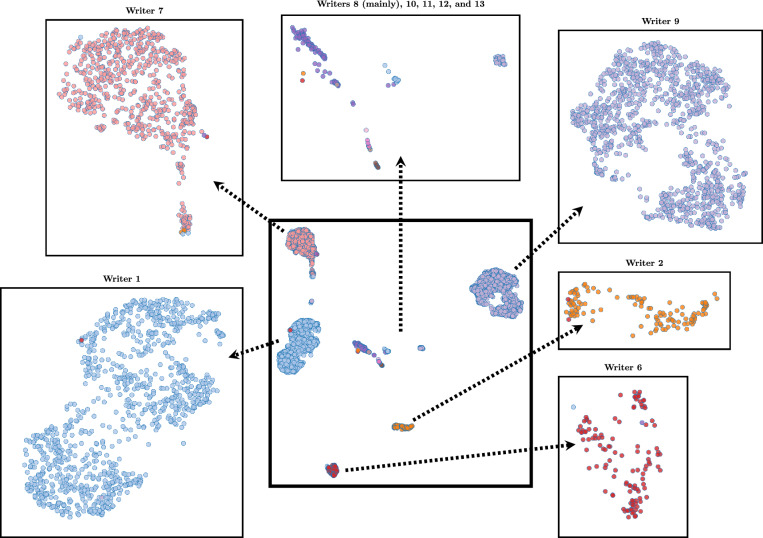
Visualization of dimensionality reduced global feature vectors of all books. Each sample point denotes one letter in the letterbooks and is color-coded by the specific writer label. The box in the middle gives an overview of all samples. The outgoing boxes are zoomed-in versions of writer clusters.

Figure 3 visualizes the global feature vectors with a UMap dimensionality reduction^23^. In the middle, the total view of the outputs is given. Each sample point represents a letter of the dataset written by a specific writer that is color-coded. Around the middle figure, the most prominent clusters are zoomed in to show how clean the predictions are. The top box is used for the underrepresented clusters. The intra-class distance was still as expected, but the inter-class distance was much lower than for the most frequent writers. Upon closer inspection of the outliers, the experts recognized that it was not a single writer but often multiple writers who created the misclassified letters. Overall, the labels for the main writers are still correct; however, the issue lies in the automatic approach, which in these cases focuses on the wrong parts of the letter.

## Data Availability

Code of the semi-automatic data preparation and for loading the data and rerunning the experiments is publicly available: • Data Preparation: https://github.com/M4rt1nM4yr/letterbooks_data_preparation• Handwritten Text Recognition: https://github.com/M4rt1nM4yr/letterbooks_text_verification• Writer Identification: https://github.com/M4rt1nM4yr/letterbooks_writer_verification
